# Performance Evaluation of Ultra-Thin Wearing Course with Different Polymer Modified Asphalt Binders

**DOI:** 10.3390/polym14163235

**Published:** 2022-08-09

**Authors:** Jiangmiao Yu, Yanlin Chen, Xiaopeng Wei, Niya Dong, Huayang Yu

**Affiliations:** 1 School of Civil Engineering and Transportation, South China University of Technology, Guangzhou 510006, China; 2 Xi’an Key Laboratory of Digital Construction and Management for Transportation Infrastructure, Xi’an 710000, China; 3 Guangdong Provincial Key Laboratory of Modern Civil Engineering Technology, South China University of Technology, Guangzhou 510641, China; 4 Central Fortune Creation (Canton) Roadway Technology Co., Ltd., Foshan 528000, China

**Keywords:** ultra-thin wearing course, comprehensive performance, rutting test

## Abstract

Ultra-thin wearing course (UTWC) as an asphalt overlay is widely used in pavement maintenance for extending pavement service life. Researchers focused on improving and evaluating its performance, yet few researchers compare the performance of typical UTWCs. Moreover, some traditional asphalt mixture tests are improper for UTWC due to the thicknesses of UTWC, which is thinner than the traditional asphalt overlay. This study further evaluated the advantages and disadvantages of typical UTWCs. A series of tests were conducted to compare the comprehensive performance of three typical UWTC products, including SMA-10, Novachip-B, and GT-10. Moreover, this study improved the rutting test to evaluate its rutting performance more accurately. Rutting specimens of 20 mm thick and 50 mm thick composite specimens (20 mm UTWC + 30 mm Portland cement concrete slabs) were prepared. Two types of PCC slabs were used, including unprocessed PCC slabs and PCC slabs with preset cracks. The test results showed that Novachip-B showed the best water stability and weakest raveling resistance, while GT-10 showed the best fatigue and anti-skid performance. The rutting performance of UTWCs was reduced because of the influence of preset cracks. The rutting depth of GT-10 was only 60–90% of that of others, showing the comprehensive performance of GT-10 was better than that of others. These results provide a significant reference for the research and application of UTWC.

## 1. Introduction

Most pavements exhibit the diseases of cracking, loosening, and deformation after 3–5 years of serving, which leads to pavement service life reduction [1,2,3,4]. Researchers have contributed to developing preventive pavement maintenance treatments to extend pavement service life [5,6,7,8]. So far, one of the widely applied preventive maintenance treatments is ultra-thin wearing course (UTWC). It is an asphalt overlay that improves pavement-surface smoothness and anti-skid performance, as well as reducing noise and repairing mild to moderate pavement diseases such as mild cracks, slight loosening, and rutting (less than 15 mm) [9,10,11]. It is economic due to its thickness being usually less than 25 mm: compared with the thickness of traditional asphalt overlay pavement (40–50 mm), UTWC saves 30–40% on material costs [12,13]. In the 1970s, UTWC was firstly applied to maintain pavements in France [14]. Later, UTWCs were widely used in many countries such as Germany, the United States, the United Kingdom, and China [15,16,17,18].

With the development of UTWC, it has been widely studied by scholars. They improved UTWC performance using high-performance asphalt binders. For example, styrene-butadiene-styrene (SBS) modified asphalt, crumb rubber modified asphalt, and high-viscosity and high-elasticity modified asphalt have been widely used in UTWC. These high-performance asphalt binders improve the high-temperature performance, raveling resistance, and crack resistance of UTWC [11,19,20]. Moreover, some researchers focused on the influence of gradation on UTWC performance. Skeleton pattern gradation helped improve the high-temperature rutting resistance and anti-skid performance [3,21]. Researchers also added basalt fiber to improve the crack resistance and raveling resistance of UTWC [22]. In addition, SMA-10, Novachip-B, and GT-10 gradation were widely used in UTWC and found to have excellent comprehensive performance [15,16,18,23,24]. SMA-10 is commonly used in Germany, Novachip-B is one of the earliest UTWCs proposed by the United States, and GT-10 is a new type of UTWC widely used in China. The thickness of SMA-10, Novachip-B, and GT-10 is usually 15–25 mm, 20–25 mm, and 10–20 mm, respectively. The cost of each UTWC product varies slightly depending on the region, and this study mainly refers to the cost of three UTWC products in China. The usual thickness of SMA-10 costs 8–9 $ per square meter, the usual thickness of Novachip-B costs about 9–10 $ per square meter, and the usual thickness of GT-10 costs about 10–11 $ per square meter. These UTWC products have been used in many pavement maintenance treatments in China, and GT-10 has received excellent evaluations from investors and users in recent years.

However, studies that have compared the UTWC performance of SMA-10, Novachip-B, and GT-10 by the laboratory test are pretty limited. The performance of UTWC, such as water stability, reveling resistance, fatigue resistance, and anti-skid performance, is usually evaluated [11,15,16,17,18,19,20,23,24,25]. Furthermore, pavement diseases, such as waves, deformations, and ruts occur after a frequent traffic load, if rutting resistance is insufficient. Therefore, most specifications require UTWC to have sufficient rutting resistance. According to the specification, the traditional rutting performance test method uses a solid rubber tire with a wheel pressure of 0.7 MPa rolls on a 300 mm × 300 mm × 50 mm specimen for a round-trip speed of 42 times/min at 60 °C for 1 h [26,27]. It is worth noting that the traditional rutting test is unsuitable for testing the rutting performance of UTWC for the following reasons. Firstly, the UTWC is thinner than the traditional rutting test specimen. Secondly, UTWC is frequently applied on old cement pavement. The effect of cement concrete structure on UTWC performance should be considered when preparing a 50 mm thick sample to test UTWC performance. Furthermore, the rutting performance of UTWC is more susceptible to lower layer impacts than typical asphalt pavements due to its thinner thickness [28,29].

Some scholars have proposed new testing methods to better test the performance of UTWC. Ding et al. [30] prepared double-layered composite specimens exhibiting a 3 + 2 cm structure. Firstly, 30 × 30 × 3 cm AC-13 mixture was prepared in a 30 × 30 × 5 cm mold, then a 2 cm thick overlay was prepared with three kinds of asphalt mixture to form a composite rutting board for the functional surface durability test. Yang et al. [31] also prepared double-layered composite specimens to study the effect of basing on the rutting performance of asphalt mixture surfaces. These specimens consisted of cement stabilized macadam slab and an asphalt surface layer having a thickness much larger than 50 mm. Cui et al. [3] prepared a 1.5 cm rutting-plate mold to simulate paving thickness and study UTWC anti-slide performance attenuation. Ge [32] simulated cement pavement with Portland cement concrete (PCC) slabs to evaluate the shear and fatigue behavior of an AC overlay on the slabs using different interlayer bonding materials in laboratory performance testing. Scholars prepared thinner rutting specimens or double-layer structures to simulate the actual situation of a UTWC. Still, no research has been conducted on the rutting performance of UTWC with thinner specimens or double-layer structures.

Based on the abovementioned, this study aims to evaluate the comprehensive performance of typical UTWCs. The three UTWC products (SMA-10, Novachip-B, and GT-10) are suitable for performance comparison due to their similar thickness and cost. A series of performance tests were conducted on the three UTWCs, including residual Marshall stability, freeze-thaw split, Cantabro, four-point beam fatigue, sand-patch method, and British pendulum number (BPN) test. In addition, an improved rutting experiment was designed to evaluate the rutting performance of UTWC accurately. Three kinds of 20 mm UTWC specimens and 30 mm PCC slabs were used to prepare composite specimens. The improved rutting test was conducted on composite specimens to investigate their rutting performance. This provides a new idea for evaluating the performance of UTWC. These results will provide a basis for evaluating the advantages and disadvantages of typical UTWC and provide a significant reference for the research and application of UTWC.

## 2. Materials and Methods

### 2.1. Materials

Two kinds of modified asphalt were used. SBS-modified asphalt (PG76-22) was used as the asphalt binder of SMA-10 and Novachip-B. The SBS content is 5%. High-viscosity and high-elasticity modified asphalt (PG100-22) is an asphalt binder of GT-10. It is a mixture of matrix asphalt, SBS polymer, compatibilizer, and coupling agent, and the dosage of SBS polymer, compatibilizer, and coupling agent were 8%, 4%, and 0.3%, respectively. PG100-22 has excellent performance on dynamic viscosity, rutting resistance, and elastic recovery. The properties of asphalt binders are shown in Table 1. The coarse aggregates used were high-quality diabase with good wearing resistance and grain shape, and the fine aggregates were limestone aggregates with hard texture, no weathering, and moderate gradation. The mineral powder was used as limestone powder.

### 2.2. Specimen Preparation

#### 2.2.1. Asphalt Mixture

**Figure 1:**
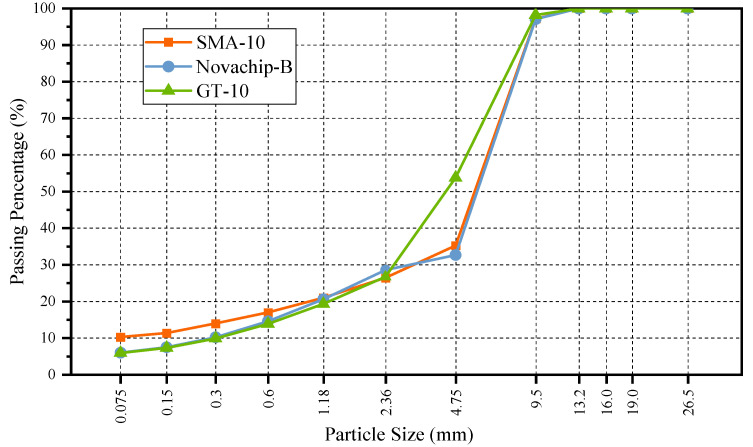
Grading curve of SMA-10-10, Novachip-B, and GT-10.

Typical gradation curves of SMA-10, Novachip-B, and GT-10 are shown in Figure 1 [18,26,33]. SMA-10 should contain 0.3% lignin fiber. The target void volume of SMA-10, Novachip-B, and GT-10 was 4, 11, and 5%, respectively. The compaction temperature of SMA-10 and Novachip-B was 165–170 °C, while for the GT-10, it was 195–200 °C. Marshall specimens were prepared for each asphalt mixture at different asphalt–aggregate ratios. Table 2 demonstrates the results. The optimum ratio for SMA-10, Novachip-B, and GT-10 was 6.4, 5, and 7.5%, respectively.

#### 2.2.2. PCC Slab with Prefab Cracks

The design strength of PCC is 40 MPa. Its main components were P.O42.5 Portland cement, medium sand, granite gravel, and water in a ratio of 1:1.62:2.43:1:0.4. The bulk densities of the Portland cement, medium sand, granite gravel, and water were 450, 728, 1092, and 180 kg/m^3^. The dimension of PCC slabs was 300 mm × 300 mm × 30 mm [32].

**Figure 2:**
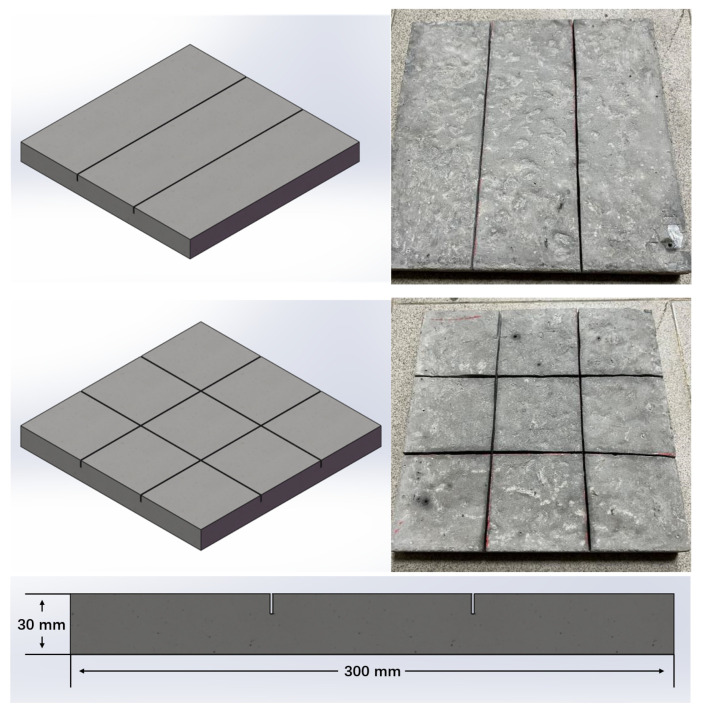
Three-dimensional (3D) view and front view of PCC slabs.

In this study, cracks were preset on 2/3 of the PCC slabs to simulate the realistic conditions of old concrete pavements, especially those with cracks and no other serious diseases. Concerning the previous findings, it is considered that the penetration type dummy joints are consistent with the actual situation of cement pavement [34,35,36,37,38,39]. The width of the preset crack set by the former researcher was between 0.5 and 3 mm, and its length was more than 300 mm, and the preset crack interval was 1/3–1/4 of PCC slab length according to other researchers. The preset crack was set as a dummy joint at a depth 1/3 of the slab thickness [40]. Therefore, cracks (1.2 mm width, 10 mm depth, 300 mm length, and 100 mm interval) were cut on PCC slabs, as shown in Figure 2. The emulsified asphalt was used to repair PCC slabs after cutting cracks [13,34,35]. In addition, to simulate the situation of cement concrete pavement with different degrees of deterioration, 2 cracks were set on 1/3 of PCC slabs, and 4 cracks were set on 1/3 of PCC slabs.

### 2.3. Testing Method

#### 2.3.1. Water Stability Test

**Figure 3:**
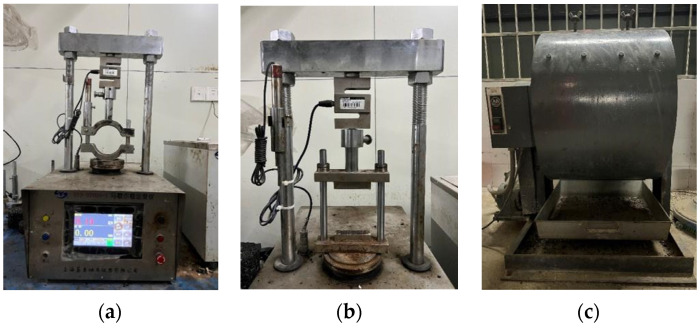
(**a**) Marshall stability test; (**b**) tensile strength test; (**c**) Cantabro test.

As Figure 3a,b shows, this study conducted immersed-Marshall and freeze–thaw tests to measure UTWC water stability. The immersed-Marshall test evaluates water damage resistance of asphalt binder, and the freeze–thaw test evaluates water stability of asphalt mixture at low temperatures or large temperature differences. The water stability test specimens are standard Marshall specimens, the size is Φ101.6 mm × 63.5 ± 1.3 mm. The number of test specimens in the same group of tests was five.

#### 2.3.2. Raveling Resistance Test

The Cantabro test was used to evaluate raveling resistance of UTWC. The Cantabro test instrument is shown in Figure 3c. The Marshall specimens were first put into the water tank (20 °C) for 20 h. After wiping the water on the surface of the specimen, it was put into the test machine to rotate for 300 cycles at a speed of 30 rpm. The raveling resistance test specimens were standard Marshall specimens, the number of test specimens in the same group of tests was five.

#### 2.3.3. Pull Strength Test

**Figure 4:**
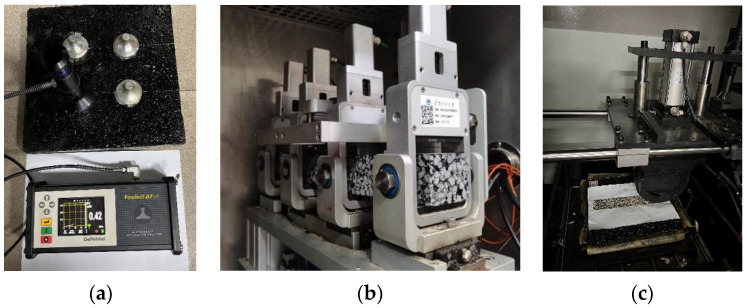
(**a**) Pull strength test; (**b**) four-point beam fatigue test; (**c**) dynamic stability test.

High-viscosity emulsified asphalt was used as a tack coat to bond three kinds of asphalt mixtures and PCC slabs. The tensile adhesion tests were conducted on three unprocessed slabs and three slabs with preset cracks, each PCC slab was tested 4 times. The amount of high-viscosity emulsified asphalt applied to the slab surfaces was 0.8 kg/m^2^ [41,42]. For the slabs with preset cracks, the test pullers were fixed to the position of cracks to test the pull strength under the most unfavorable conditions, as shown in Figure 4a.

#### 2.3.4. Fatigue Test Procedure

This study used four-point beam test to evaluate the fatigue performance of asphalt mixtures. Cooper NU-14 tester (Cooper Research Technology-Technical Centre, Ripley, Derbyshire, UK, Figure 4b) was used. The tests were performed at 15 ± 0.5 °C. The size of the fatigue test specimen is 380 ± 5 mm length, 63.5 ± 5 mm width, and 50 ± 5 mm height. The fatigue performance at strain levels of 400, 600, 800, and 1000 micro-strains was evaluated, and a loading frequency of 10 Hz was used, a standard frequency for fatigue tests. Four specimens were tested under each strain level. Failure was assumed to occur when the stiffness of the specimen reached half of its initial value. The initial value was determined by 50 loading cycles. The test was terminated automatically when this load diminished by 50% [43,44].

#### 2.3.5. Rutting Test Procedure

The rutting test of an asphalt mixture is used to evaluate its high-temperature rutting resistance. The test instrument is shown in Figure 4c. The dynamic stability (DS) and the rutting depth (RD) of 300 mm × 300 mm × 50 mm rutting specimens from the three asphalt mixtures were measured. Three specimens of each asphalt mixture were prepared for the rutting test. DS is obtained by measuring the relationship between the number of wheel loads and the deformation of slab specimens. A larger DS means better high-temperature stability. The rutting test was conducted after 5 h at a temperature of 60 °C. The wheel-driving direction was consistent with the compaction direction in the specimen molding. The deformation of the asphalt mixture after 45 and 60 min was recorded separately. The total number of round trips was divided by the gap of specimen deformation at 60 and 45 min to determine the DS value. DS can be calculated using Equation (1):

(1) $$\mathrm{DS}=\frac{\left(t_{1}-t_{2}\right)\cdot 42}{d_{1}-d_{2}}\cdot c_{1}\cdot c_{2}$$

DS—dynamic stability of asphalt mixtures;
*t*_1_,*t*_2_—test time, usually 45 min and 60 min;
*d*_1_,*d*_2_—rutting depth of specimen surface corresponding to t1 and t2 at test time, mm;
*c*_1_,*c*_2_—correction factors;

#### 2.3.6. Improved Rutting Test

This study designed an improved rutting test. A UTWC specimen with a dimension of 300 mm × 300 mm × 20 mm was used for the rutting test. The rutting specimens usually need to be compacted several times with compaction machines and the compaction time of UTWC was different from the 50 mm-thick specimen used in the traditional rutting test. The test set compaction times as 24, 20, 16, and 12, ensuring UTWC specimens meet compaction standard. A steel plate, dimensions 300 mm × 300 mm × 30 mm, was placed in the rutting mold. Viscosity-modified emulsified asphalt was spread on a steel plate, amounting to 0.8 kg/m^2^. Then, the asphalt mixture was compacted into the UTWC specimen under different compaction times with a volume of 1800 cm^3^ (30 cm × 30 cm × 2 cm) placed on the steel plate according to the bulk density of different asphalt mixtures.

**Figure 5:**
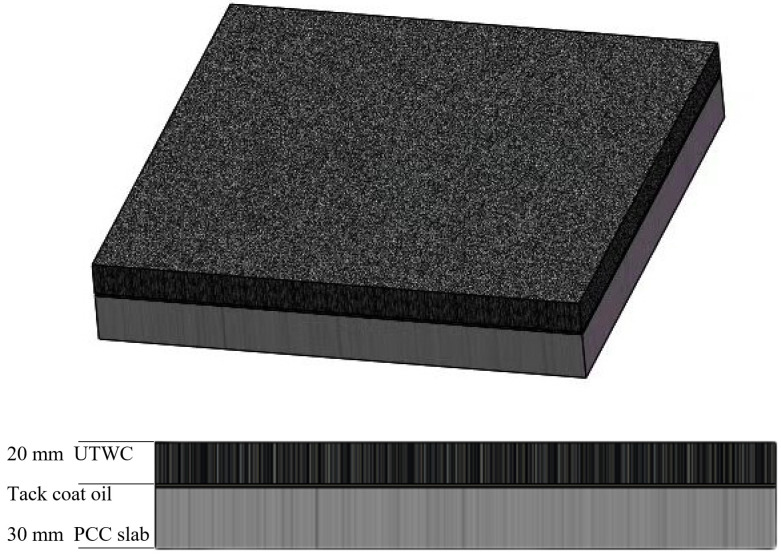
The 3D view and front view of the composite specimen.

The asphalt mixture was also compacted on a PCC slab to form a composite specimen to simulate the actual situation of overlaying UTWC onto old cement pavement. As shown in Figure 5, the composite specimens from top to bottom were UTWC, tack coat oil of emulsified asphalt, and the PCC slab. Rutting tests were conducted on the specimens according to the methods described in Section 2.3.5. Three 20 mm UTWC specimens and three composite specimens of each UTWC were prepared for the improved rutting test.

#### 2.3.7. Anti-Skid Performance and Durability Test

**Figure 6:**
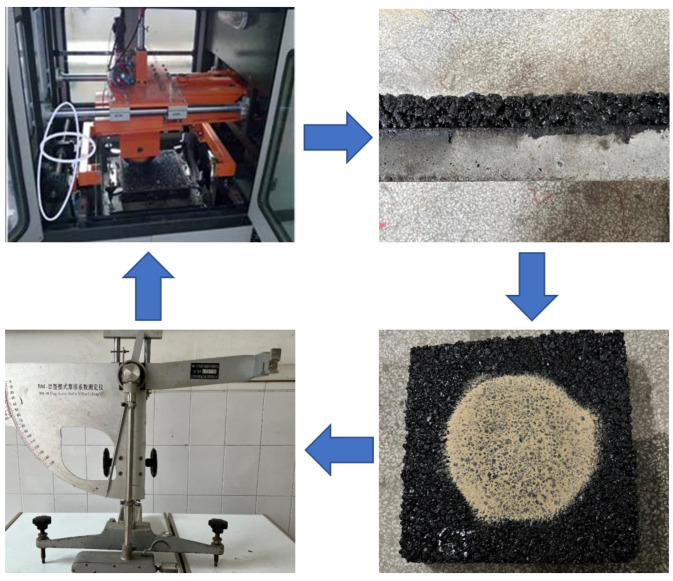
Kneading test process.

The anti-skid performance and durability of UTWC were evaluated based on the loading kneading tester. We added a lateral moving motor on the rutting test machine to make W-shaped movement possible, stimulating the change of asphalt pavement surface structure under the action of a moving tire. This process is called the kneading test [45]. The latitude speed of the wheel is 10 cm/min, the longitude speed is 42 ± 1 times/min, and the weight of the kneading wheel is 42–100 kg (adjustable). The loading and environmental simulations of this acceleration load kneading machine are practical, and it is easy to operate. The total kneading time of each specimen was 8 h divided into 5 stages, 0, 2, 4, 6, and 8 h. The sand-patch method and a BPN test at room temperature (25 ± 1 °C) were used to measure the attenuation pattern of the surface anti-skid performance, with 2 h testing interval. Three specimens of each UTWC were prepared for the kneading test. The test process is shown in Figure 6. The durability of a UTWC was visually measured by observing the fracture.

## 3. Results and Discussion

### 3.1. Water Stability

The residual Marshall test and freeze–thaw test were used in this study to reflect the ability of an asphalt mixture to resist failure caused by asphalt membrane peeling and particle drop. The residual Marshall mainly evaluates the water stability of the asphalt mixture at high temperatures, and the freeze–thaw test mainly evaluates its water stability at low temperatures or at high temperature differences. The water stability of asphalt mixtures can be better evaluated by using both tests. Although the freeze–thaw test has a freeze–thaw cycle, it is also applicable to non-freezing areas because it is designed to evaluate water stability.

**Figure 7:**
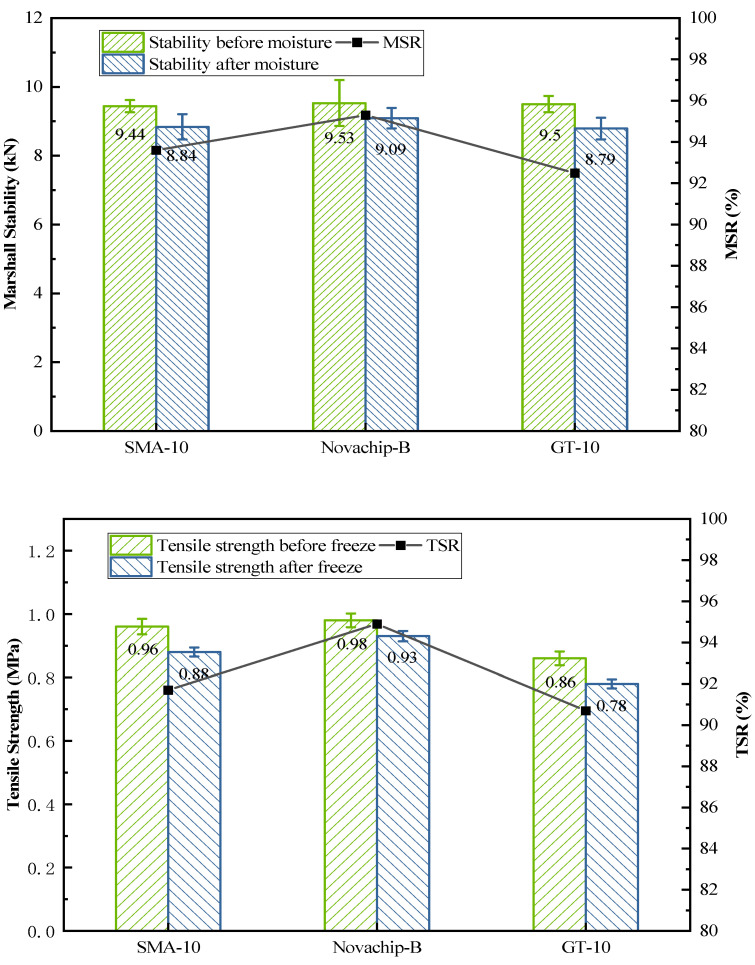
Results of Marshall stability and tensile strength.

Table 3 and Figure 7 show that the Marshall stability of the three asphalt mixtures was about 9.5 kN. The Marshall stability of SMA-10, Novachip-B, and GT-10 was also very close even after immersion, which was 8.84, 9.09, and 8.79, respectively. The residual Marshall stability of SMA-10, Novachip-B, and GT-10 were 93.6, 95.3, and 92.5%, respectively. Table 4 and Figure 7 show the tensile strength of the asphalt mixture before and after the freeze-thaw. Afterward, the tensile strength ratio of SMA-10 Novachip-B and GT-10 was 91.7, 94.9, and 90.7%, respectively, which met the technical requirements of at least 80%. Noticeably, the residual stability of the three mixtures was above 90%. The same results also appeared in the freeze–thaw test, indicating that the water stability of the mixtures as typical UTWC engineering products was excellent.

### 3.2. Raveling Resistance

When under repeat traffic, insufficient asphalt dosage and asphalt bonding cause aggregate shedding and scattering, which further leads to pavement potholes. This is a common disease in UTWC. It is necessary to supplement the UTWC performance testing with a raveling resistance test to prevent this damage. The raveling loss is an indicator of the asphalt mixture raveling resistance, measured by the Cantabro test and expressed as a percentage. The smaller the raveling loss, the better the raveling resistance of the asphalt mixture.

Table 5 shows that the raveling loss of SMA-10, Novachip-B, and GT-10 was 4.11, 6.38, and 4.93%, respectively, and did not exceed 8% according to the specifications. The difference between the raveling loss of SMA-10 and GT-10 is not large, but the raveling loss of Novachip-B is nearly 2% larger than the raveling loss of SMA and GT-10, indicating that Novachip-B had the worst raveling resistance. SMA-10 and Novachip-B asphalt mixtures both underwent gap gradation, but the air void of Novachip-B was larger than SMA-10, and the film thickness was lower than SMA-10. Therefore, the raveling resistance of SMA-10 was better than that of Novachip-B. GT-10 had a dense skeleton structure, larger asphalt film thickness, and better raveling resistance than Novachip-B.

### 3.3. Pull Strength

The pull strength of high-viscosity emulsified asphalt used in this study was thoroughly studied by the former research group. The pull strength should be higher than 0.4 MPa to ensure UTWC is closely bonded with the original cement concrete pavement [11,12,18]. The disease caused by insufficient bond strength did not occur when high-viscosity emulsified asphalt pull strength was more than 0.4 MPa. A few researchers applied this high-viscosity emulsified asphalt on PCC slabs with preset cracks for pull strength tests before. To ensure the applicability of high-viscosity emulsified asphalt, the pull strength of high-viscosity emulsified asphalt was measured by the tensile adhesion test. As Table 6 shows, the pull strength of high-viscosity emulsified asphalt on the unprocessed PCC slab interface was 0.49 MPa, and for PCC slabs with 2 and 4 preset cracks, 0.45 and 0.42 MPa, respectively. Even in the most unfavorable position, the pull strength reached the specification requirements of more than 0.4 MPa. Therefore, high-viscosity emulsified asphalt can be used as a tack coat on PCC slabs with preset cracks. It ensures the mechanical occlusion effect between the asphalt mixture and cement concrete. Thus, the pull strength of the high-viscosity emulsified asphalt ensures the reliability of experimental results, including the 20 mm specimens and the composite specimens.

### 3.4. Fatigue Resistance

UTWC asphalt overlay is subjected to repeated vehicle loading. It fails when the repeated loadings exceed its designed fatigue life. Therefore, UTWC requires longer fatigue life. Fatigue resistance refers to the ability of the asphalt mixture to withstand repeated loadings. The better the fatigue resistance, the longer the fatigue life.

**Figure 8:**
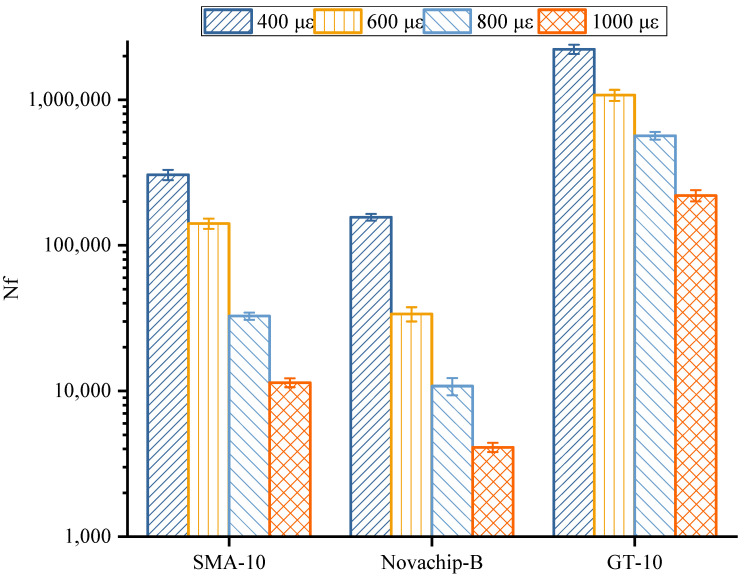
Results of four points bending fatigue test.

The differences obtained for different asphalt mixtures are shown in Figure 8 and Table 7. At each micro-strain, the fatigue life of the asphalt mixture is the largest in GT-10, followed by SMA-10 and the smallest in Novachip-B. At lower strain levels (<600 micro-strains), the fatigue life of GT-10 was higher than 1 million cycles. For higher strain levels (>800 micro-strain), GT-10’s fatigue life was almost 18 times higher than that of SMA-10 and nearly 53 times higher than that of Novachip-B. The higher the strain level, the more significantly increased proportion of the fatigue life of GT-10 to the fatigue of the other two mixtures. Therefore, the GT-10 asphalt mixture exhibited better fatigue resistance at higher strain levels (800–1000 micro-strain). The better fatigue resistance was mainly due to the high PG grade asphalt binder and thicker asphalt film, which significantly improved the toughness of the GT-10. In addition, it was not easy to peel under repeated loads, thus significantly enhancing the fatigue resistance of the mixture.

### 3.5. General Rutting Test Performance

Insufficient rutting resistance leads to pavement diseases, such as waves, deformations, and ruts occurring after a frequent traffic load. The better rutting resistance of the asphalt mixture, the better the rutting performance, and a larger DS and a smaller RD represent better rutting performance.

**Figure 9:**
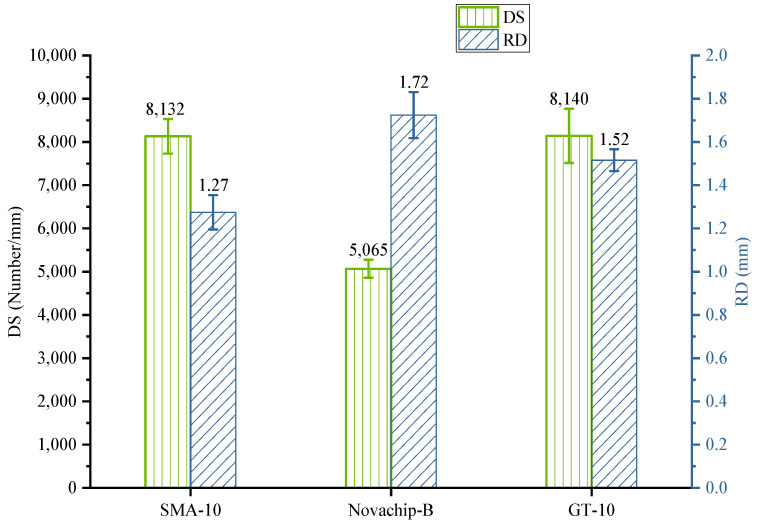
Results of general rutting test.

The results of general rutting performance experiments are shown in Figure 9. The DS of the three asphalt mixtures met the specification of higher than 3000 times/mm. The DS of SMA-10 and GT-10 was 8132 and 8140 times/mm, respectively. The DS of SMA-10 and GT-10 was about 60% higher than that of Novachip-B, indicating their better rutting performance. Meanwhile, the rutting depth (RD) of SMA-10 was 1.277 mm, only 84.1 and 73.9% of GT-10 and Novachip-B, respectively. Overall, SMA-10 had the best rutting performance, followed by the GT-10 and Novachip-B.

### 3.6. Improved Rutting Test Performance

#### 3.6.1. Compaction Times of UTWC

The compaction times of traditional rutting test specimens are usually set to 24. However, Ding et al. [30] mentioned that UTWC compaction times should be considered separately. The test set different compaction times (from high to low 24, 20, 16, 12) to ensure that the 20 mm UTWC specimens met the compaction standard. The drill core specimen was taken after the specimen was compacted, and the bulk density was measured. The bulk-density test method was consistent with the Marshall specimen to reduce the error. When the bulk density reaches 100% ± 1 of the same Marshall specimen bulk density, the 20 mm UTWC specimens can meet the compaction standard.

**Figure 10:**
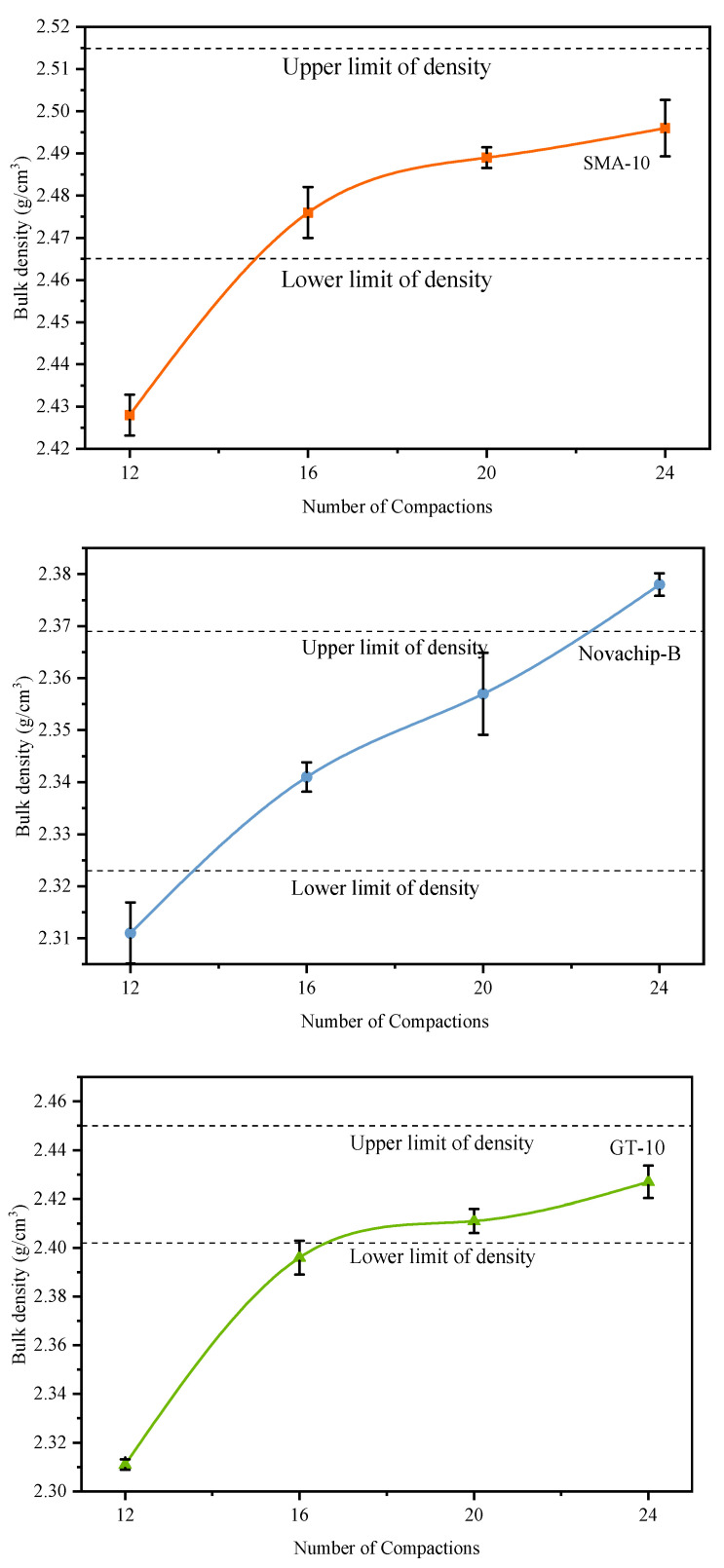
The bulk density of UTWC specimens.

As shown in Figure 10, the bulk density of UTWC specimens continued to increase with the compaction times. The bulk density increased rapidly in the early stage and gradually stabilized in the latter. When the compaction times were 18 ± 1, the specimen bulk density of SMA-10 and Novachip-B was close to the median of the allowable region. When the compaction time was 24, the bulk density of the GT-10 was close to the median acceptable region because of fine gradation and high-viscosity and high-elasticity-modified asphalt. This indicated that the GT-10 required greater compaction force, whereas SMA-10 and Novachip-B were easier to compact. Therefore, the compaction times of SMA-10 and Novachip-B specimens were set to 18, and GT-10 specimens were set to 24.

#### 3.6.2. Rutting Performance of Composite Specimen

**Figure 11:**
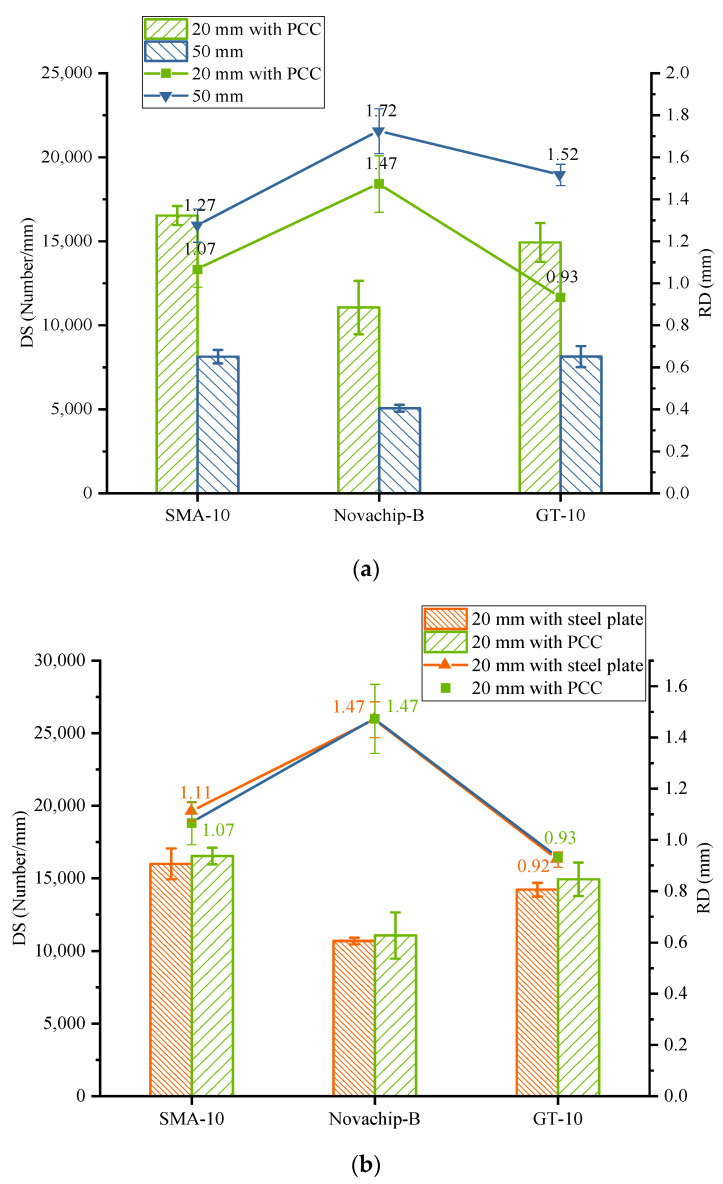
Rutting results: (**a**) UTWC specimens of 50 mm and 20 mm with PCC slab; (**b**) UTWC specimens of 20 mm with steel plate.

The test results of a 20 mm UTWC formed on an unprocessed PCC slab and a 50 mm rutting test specimen are shown in Figure 11a. It shows that the RD of all kinds of mixtures decreased while the DS increased. The RD of SMA-10 and Novachip-B decreased by 16.4 and 14.6%, while DS increased by 103 and 118%, respectively. The DS of GT-10 increased by 83%, and the RD decreased by 38.5%. The RD of GT-10 was also the lowest of the three asphalt mixtures. The RD of the 20 mm UTWC specimen is not so low as to be negligible. Therefore, the rutting test results of the 20 mm UTWC specimen are reliable.

The RD and DS of the UTWC specimens formed on steel plates and unprocessed PCC slabs are shown in Figure 11b. The DS and RD of the two different 20 mm UTWC specimens are very close for SMA-10 and Novachip-B with gab gradation and GT-10 with dense gradation. The deviation values of all results are within 3%. The test results of two different specimens may be equivalent in the rutting test, considering the experimental error because the unprocessed PCC slab with no crack can be regarded as rigid as a steel plate in forming UTWC specimens. Therefore, consistent experimental results for the rutting test can be obtained when UTWC specimens were prepared on the unprocessed PCC slab and steel plate using the same quality of asphalt mixture, the same temperature, and the number of compaction times.

**Figure 12:**
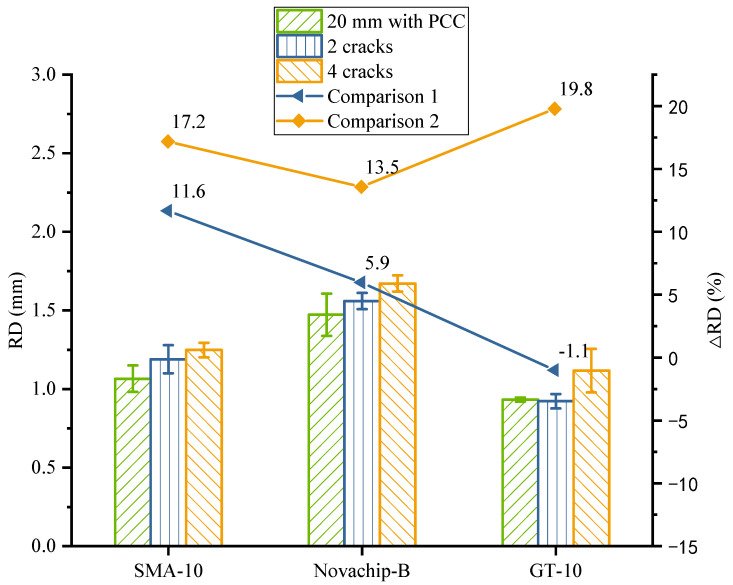
Results of composite specimens with different cracks.

The rutting test results of composite specimens with two and four preset cracks are shown in Figure 12 and Table 8. The growth between the RD of the composite specimen with two preset cracks and the one with the unprocessed PCC slab was expressed by the Comparison 1 virtual line. The growth between the RD of the composite specimen with four preset cracks and the RD with the unprocessed PCC slab is expressed by the Comparison 2 virtual line. From the Comparison 1 virtual line, the RD increase of SMA-10 and Novachip-B reached 11.6 and 5.9%, and the results of the RD had obvious changes. The RD of GT-10 was reduced by about 1.1%. Considering experimental error, the DS of the two GT-10 specimens was similar, so its RD did not change. The rutting performance of GT-10 was not affected by the two preset cracks, and its RD was only 77.6% of that of SMA-10 and 59.2% of that of Novachip-B. From the Comparison 2 virtual line, the RD changes in the three mixtures were undeniable. The stress concentration occurred at the preset crack of PCC slab in the rutting test, so that the bottom and surface of the local UTWC specimen were subjected to greater shear stress than those on the complete PCC slab. This led to the destabilization of the structure of the UTWC due to shear damage, resulting in a larger RD [46]. The RD increase in SMA-10, Novachip-B, and GT-10 reached 17.2, 13.5, and 19.8%, respectively. However, it is worth noting that the RD of GT-10 was the smallest, and its DS the largest. Meanwhile, the RDs of SMA-10 and Novachip-B were close to that of the 50 mm specimen. In contrast, the RD of GT-10 was only 73.7% of the RD of the 50 mm specimen. The RD of GT-10 was only 89.5% of that of SMA-10 and 66.8% of that of Novachip-B. GT-10 had the better rutting performance even though the number of preset cracks increased.

As SMA-10 and Novachip-B underwent gap gradation, the same SBS-modified asphalt was used for the two mixtures, the variation patterns of which were relatively close. GT-10 had dense gradation with high-viscosity and high-elasticity modified asphalt. Therefore, the variation pattern of GT-10 was different and obtained a better rutting performance than from SMA-10 and Novachip-B.

In summary, the rutting performance of UTWC decreased due to the effect of pre-determined cracks, and the range of decrease varied with different types of UTWC. Compared with the composite specimens on unprocessed PCC slabs, the RD of SMA-10, Novachip-B, and GT-10 composite specimens with two and four preset cracks increased by 11.6, 5.9, and −1.1%, and 17.2, 13.5, and 19.8%, respectively. The RD of GT-10 was 60–90% of that of the others. Therefore, the combined rutting performance of GT-10 is better than others. Notably, the increase in RD and decrease in rutting performance for the three asphalt mixtures reveal a situation. This situation is in line with the fact that the actual rutting performance of UTWC in practical applications is weaker than the design rutting performance. The service life of UTWC is affected by insufficient rutting performance, resulting in the actual service life (usually 3–4 years) of the UTWC being half or less than the design life (usually 8–10 years) [47,48]. The results indicated that compared with the traditional rutting test, the improved rutting test predicts the actual rutting performance of UTWC more accurately to some extent.

### 3.7. Anti-Skid Performance and Durability

**Figure 13:**
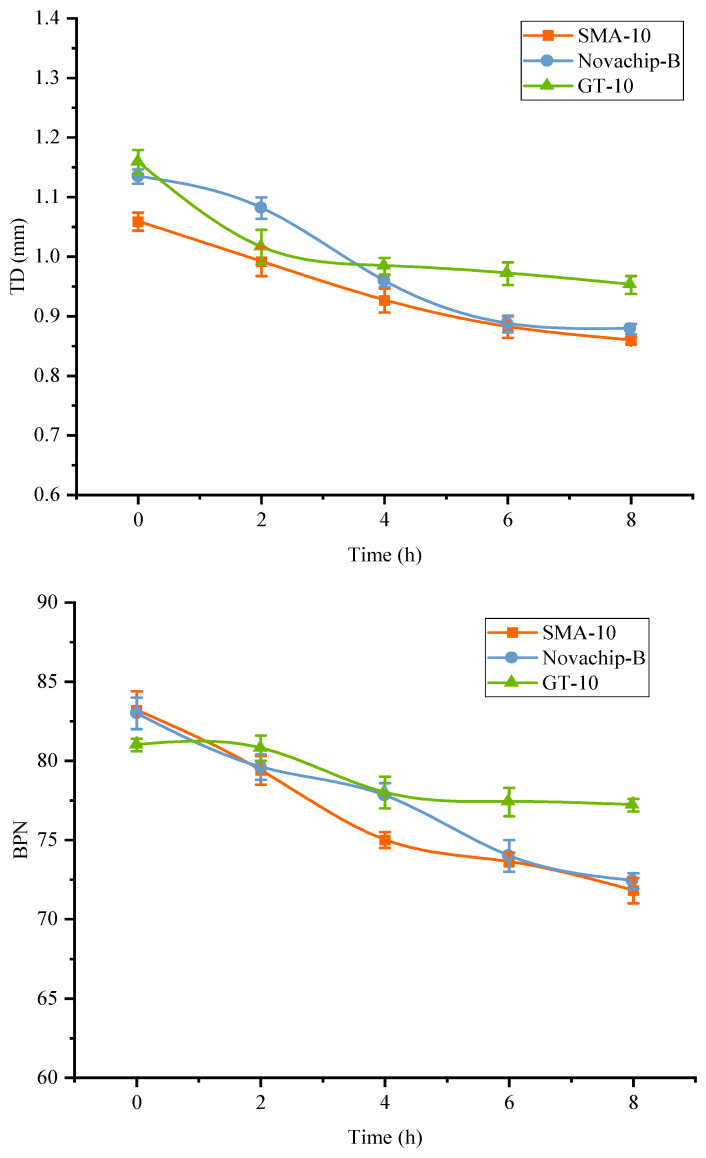
Results of the anti-skid performance test.

The anti-skid performance test effectively reflects skid resistance attenuation, and it was characterized by the texture depth (TD) and BPN in this study. As shown in Figure 13, as kneading times increased, the BPN and TD of the three UTWCs presented an obvious downturn, and the corresponding anti-skid performance decreased. After the kneading experiment at room temperature for 8 h, the TD of SMA-10, Novachip-B, and GT-10 decreased by 18.9, 22.6, and 17.8%, respectively. In contrast, the BPN decreased by 13.7, 12.8, and 4.7%. The TD and BPN of GT-10 were also superior to the other two mixtures in absolute value. However, the TD and BPN of each exhibited different variation patterns. GT-10 decreased with a fast attenuation rate in the early stage, but then it slowed down and stabilized in the later stage. For SMA-10 and Novachip-B, the TD and BPN decreased continuously, and the attenuation rate did not slow down. It can be concluded that the anti-skid performance of GT-10 was superior to that of Novachip-B and SMA-10.

Table 9 shows that, after an 8 h kneading experiment at room temperature, the three mixtures were unchanged with no apparent cracks or rutting, showing that three kinds of asphalt mixtures had good durability at room temperature.

### 3.8. Comprehensive Performance

Based on the results and discussions, the performances of three typical UTWCs are shown in Table 10, including water stability, raveling resistance, fatigue resistance, anti-skid performance, durability, and rutting performance based on general rutting test and improved rutting test. Among the three UTWCs, Novachip-B showed the best water stability, SMA-10 showed the best raveling resistance, and GT-10 was the best in fatigue resistance, anti-skid performance, and durability. GT-10 and Novachip-B showed similar performance in the water stability, and the gap between the raveling resistance of GT-10 and that of SMA-10 was minimal. However, the advantage of GT-10 in fatigue resistance and anti-skid performance was more prominent. In particular, in the four-point beam fatigue test at higher strain levels, GT-10 exhibited stronger fatigue life than SMA-10 and Novachip-B.

Moreover, the rutting performance of GT-10 was the best in the improved rutting tests. Comparing the test results, the rutting test of 20 mm specimens and two different composite specimens showed nice agreement, and the rutting performance of the three UTWCs was ranked the same. SMA-10 showed the best rutting performance in the general rutting test, which was not consistent with the results of the improved rutting test. Therefore, the improved rutting test is recommended to evaluate the rutting performance of UTWC for an asphalt overlay with a thickness of nearly 20 mm. GT-10 showed the best comprehensive performance among the three typical UTWC products.

## 4. Conclusions

This study compared the comprehensive performance of three typical UTWC products of similar thickness and cost. Typical gradations and asphalt–aggregate ratios for each product were used to prepare UTWC specimens. This study conducted a series of laboratory tests on UTWCs to investigate the differences in their water stability, raveling resistance, fatigue resistance, anti-skid performance, and durability. The water stability of UTWC was investigated by the residual Marshall stability and freeze–thaw split test. Then, the Cantabro test and four-point beam fatigue test were conducted to get the raveling resistance and fatigue resistance of UTWC. Furthermore, an improved rutting test was designed in this study, and rutting tests were applied to 15 different specimens to investigate the rutting performance of UTWC. Finally, the anti-skid performance of UTWC was evaluated by the sand-patch method test and BPN test, and the durability was investigated by observing the changes of several UTWCs during the 8 h kneading test. According to the experimental results, the following conclusions can be drawn:

- The water stability of all kinds of UTWCs was excellent. Novachip-B showed weaker raveling resistance than others, while GT-10 showed the best fatigue resistance and anti-skid performance.
- The recommended compaction times for 20 mm SMA-10, Novachip-B, and GT-10 specimens were 18, 18, and 24, respectively. The rutting performance of all specimens on the steel plate and unprocessed PCC slab was similar, which was considered equivalent under specific circumstances.
- The rutting performance of UTWCs was reduced because of the influence of preset cracks, and different UTWCs were affected to various degrees. Compared with the unprocessed PCC slab specimens, the RD of SMA-10, Novachip-B, and GT-10 composite specimens with two and four preset cracks increased by 11.6, 5.9, and −1.1%, and 17.2, 13.5, and 19.8%, respectively. The improved rutting test predicts the actual rutting performance of UTWC more accurately.
- GT-10 showed the best comprehensive performance among the three typical UTWC products.

The study evaluated the comprehensive performance of typical UTWCs, from water stability to rutting performance. The results provide a reference for the research and development of UTWC performance. However, the longer test time of UTWC rutting test could be considered in the future. The durability of the UTWCs and the anti-reflection crack performance of UTWCs will be investigated by rutting test machine or other equipment that can provide rutting loads based on the conclusions of this paper. Moreover, the fatigue performance of composite specimens will be further studied by the four-point beam test, which is essential for evaluating the comprehensive performance of UTWC.

## Figures and Tables

**Table 1:** Properties of the SBS modified asphalt (PG76-22) and high-viscosity and high-elasticity modified asphalt (PG100-22).

Properties	Units	Technical Requirement		Test Results	
		PG76-22	PG100-22	PG76-22	PG100-22
Penetration (25 °C, 5 s, 100 g)	0.1 mm	40–60	30–50	53	38
Softening point	°C	≥75	≥95	89	98
Elastic recovery (25 °C)	%	≥90	≥98	96	99.5
Solubility (Trichloroethylene)	%	≥99	≥99	99.8	99.8
Storage stability	°C	≤2.0	≤2.5	1.2	2.1
After short-term aging					
Mass loss	%	±1.0	±1.0	−0.014	+0.01
Penetration ratio (25 °C)	%	≥65	≥70	78.1	83.9
G*/sin δ 2.2 kPa critical temperature	°C	≥76	≥100	76.6	100.9

**Table 2:** Volume index under different asphalt–aggregate ratios.

Asphalt–Aggregate Ratio (%)		Porosity (%)	VMA (%)	VFA (%)	VFAmix (%)	Asphalt Film Thickness (μm)	Rate of Run-Off Loss (%)
SMA-10	6.1	4.6 ± 0.2	17.0 ± 0.1	73.0 ± 0.6	36.7 ± 0.2	11.74	0.09 ± 0.01
	6.4	3.9 ± 0.1	17.1 ± 0.1	76.9 ± 0.2	36.6 ± 0.2	12.8	0.12 ± 0.01
	6.7	3.3 ± 0.1	17.1 ± 0.1	80.7 ± 0.7	36.4 ± 0.1	13.02	0.17 ± 0.03
Novachip-B	4.7	12 ± 0.1	20.8 ± 0.1	42.2 ± 0.3	40.5 ± 0.1	8.71	0.05 ± 0.01
	5.0	11.2 ± 0.4	20.7 ± 0.3	45.8 ± 0.8	40.2 ± 0.3	9.35	0.06 ± 0.2
	5.3	10.4 ± 0.6	20.5 ± 0.3	49.4 ± 1.6	39.9 ± 0.5	9.99	0.11 ± 0.02
GT-10	7.2	5.5 ± 0.2	20 ± 0.1	72.7 ± 0.7	38.2 ± 0.1	14.16	0.22 ± 0.03
	7.5	5.1 ± 0.3	20.2 ± 0.1	74.7 ± 1.1	38.2 ± 0.3	14.79	0.27 ± 0.03
	7.8	4.6 ± 0.2	20.3 ± 0.5	77.5 ± 2.2	38.1 ± 0.4	15.43	0.33 ± 0.03

**Table 3:** Results of Marshall stability test.

Mixture Type	Marshall Stability (kN)		Residual Marshall Stability (%)
	60 °C, 0.5 h	60 °C, 48 h	
SMA-10	9.44 ± 0.31	8.84 ± 0.51	93.6
Novachip-B	9.53 ± 1.09	9.09 ± 0.40	95.3
GT-10	9.50 ± 0.42	8.79 ± 0.40	92.5

**Table 4:** Results of tensile strength test.

Mixture Type	Unfreeze-Thaw Group		Freeze-Thaw Group		Tensile Strength Ratio (%)
	Critical Load (kN)	Tensile Strength (MPa)	Critical Load (kN)	Tensile Strength (MPa)	
SMA-10	9.56 ± 0.53	0.96 ± 0.06	8.86 ± 0.30	0.88 ± 0.03	91.7
Novachip-B	9.84 ± 0.55	0.98 ± 0.06	9.36 ± 0.35	0.93 ± 0.03	94.9
GT-10	8.71 ± 0.35	0.86 ± 0.03	7.85 ± 0.32	0.78 ± 0.03	90.7

**Table 5:** Cantabro test results.

Mixture Type	Before Testing (g)	After Raveling (g)	Raveling Loss (%)
SMA-10	1216.9 ± 16.8	1166.9 ± 9.6	4.11
Novachip-B	1203.4 ± 11.4	1126.6 ± 9.7	6.38
GT-10	1224.9 ± 6.9	1164.5 ± 5.6	4.93

**Table 6:** Pull strength test results.

PCC Type	Pull Strength/MPa
Unprocessed PCC slab	0.49 ± 0.02
PCC slab with 2 cracks	0.45 ± 0.03
PCC slab with 4 cracks	0.42 ± 0.02

**Table 7:** Results of four points bending fatigue test.

Mixture Type	Cycles of Failure			
	με = 400	με = 600	με = 800	με = 1000
SMA-10	305,368 ± 32,282	141,203 ± 15,562	32,684 ± 2419	11,404 ± 1053
Novachip-B	155,874 ± 10,274	33,841 ± 5179	10,801 ± 1848	4104 ± 419
GT-10	2,224,587 ± 215,535	1,075,369 ± 78,377	567,031 ± 48,516	220,015 ± 25,905

**Table 8:** Results of composite specimens.

Mixture Type	PCC Slab	Rutting Depth (mm)	DS (number/mm)	Amplification of RD (%)
SMA-10	Two cracks	1.1895 ± 0.1	14,691 ± 731	11.6
	Four cracks	1.2484 ± 0.053	9214 ± 432	17.2
Novachip-B	Two cracks	1.5597 ± 0.054	9144 ± 329	5.9
	Four cracks	1.6719 ± 0.053	5880 ± 393	13.5
GT-10	Two cracks	0.9226 ± 0.05	14,414 ± 953	−1.1
	Four cracks	1.1169 ± 0.11	10,674 ± 788	19.8

**Table 9:** The situation of composite specimens every 2 h.

Time	SMA-10	Novachip-B	GT-10
0 h	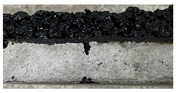	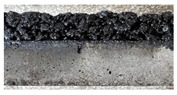	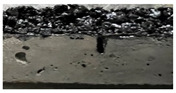
2 h	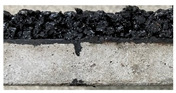	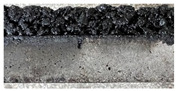	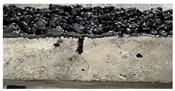
4 h	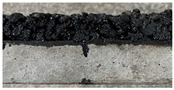	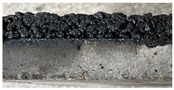	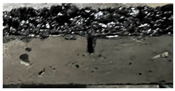
6 h	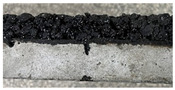	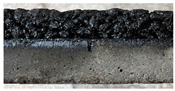	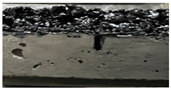
8 h	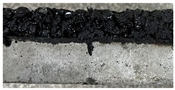	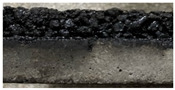	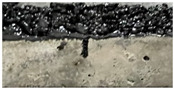

**Table 10:** Results of UTWC comprehensive performance.

Performance		Mixture Type		
		SMA-10	Novachip-B	GT-10
Water stability		2	1	3
Raveling resistance		1	3	2
Fatigue resistance		2	3	1
Anti-skid performance		3	2	1
Durability		1	1	1
General rutting performance		1	3	2
Improved rutting performance	20 mm specimen	2	3	1
	composite specimen with 2 cracks	2	3	1
	composite specimen with 4 cracks	2	3	1

In the same experiment, 1 represents excellent, 2 represents medium, and 3 represents weak.

## Data Availability

The data presented in this study are available on request from the corresponding author.
